# Menopause influences lung inflammatory response and miRNA expression in brain death donors

**DOI:** 10.3389/ti.2026.16846

**Published:** 2026-09-04

**Authors:** Elizabeth Cristina Miola, Nicholas Pietro Agulha Toneto, Fernanda Yamamoto Ricardo-da-Silva, Pedro Luis Zonta de Freitas, Marina Vidal-dos-Santos, Luciana Coelho Marques, Henri Gerrit Derk Leuvenink, Luiz Felipe Pinho Moreira, Cristiano Jesus Correia, Ana Cristina Breithaupt-Faloppa

**Affiliations:** 1 Laboratório de Cirurgia Cardiovascular e Fisiopatologia da Circulação (LIM-11), Instituto do Coração (InCor), Faculdade de Medicina USP, São Paulo, Brazil; 2 Department of Surgery, University Medical Centre Groningen, University of Groningen, Groningen, Netherlands

**Keywords:** brain death, donor, inflammation, lung, menopause, rats

## Abstract

Lungs are highly affected by brain death, with females showing a higher inflammatory response, linked sex hormones acute reduction. With the aging of world population, the number of older donors is increasing. So, the study of menopause associated changes gain importance. Here we investigated menopause’s effects in female brain death rats, previously subjected to transitional follicular depletion and aging. Female Wistar rats were divided in young and menopause groups. After follicular depletion, rats aged for 10 weeks. The animals were submitted to brain death and Sham operated rats served as controls. White blood cell counts, bronchoalveolar lavage were analyzed and inflammatory mediators were quantified. Lung tissue was evaluated for myeloperoxidase, intercellular adhesion molecules, miRNA expression, and protein and gene expression of estradiol receptors. In menopause group, there was increase in systemic and tissue leukocyte infiltration, myeloperoxidase expression, inducible nitric oxide synthase, intercellular adhesion molecule-1, lung edema, and loss of IL-10 regulation. Additionally, we found alterations in estradiol receptors along with changes in the expression of miRNAs associated to inflammation, vascular function and senescence. Menopause increases lung inflammation after brain death, by higher leukocyte infiltration and reduction of IL-10 and may impact the outcome of lung transplantation.

## Graphical Abstract

**Figure:**
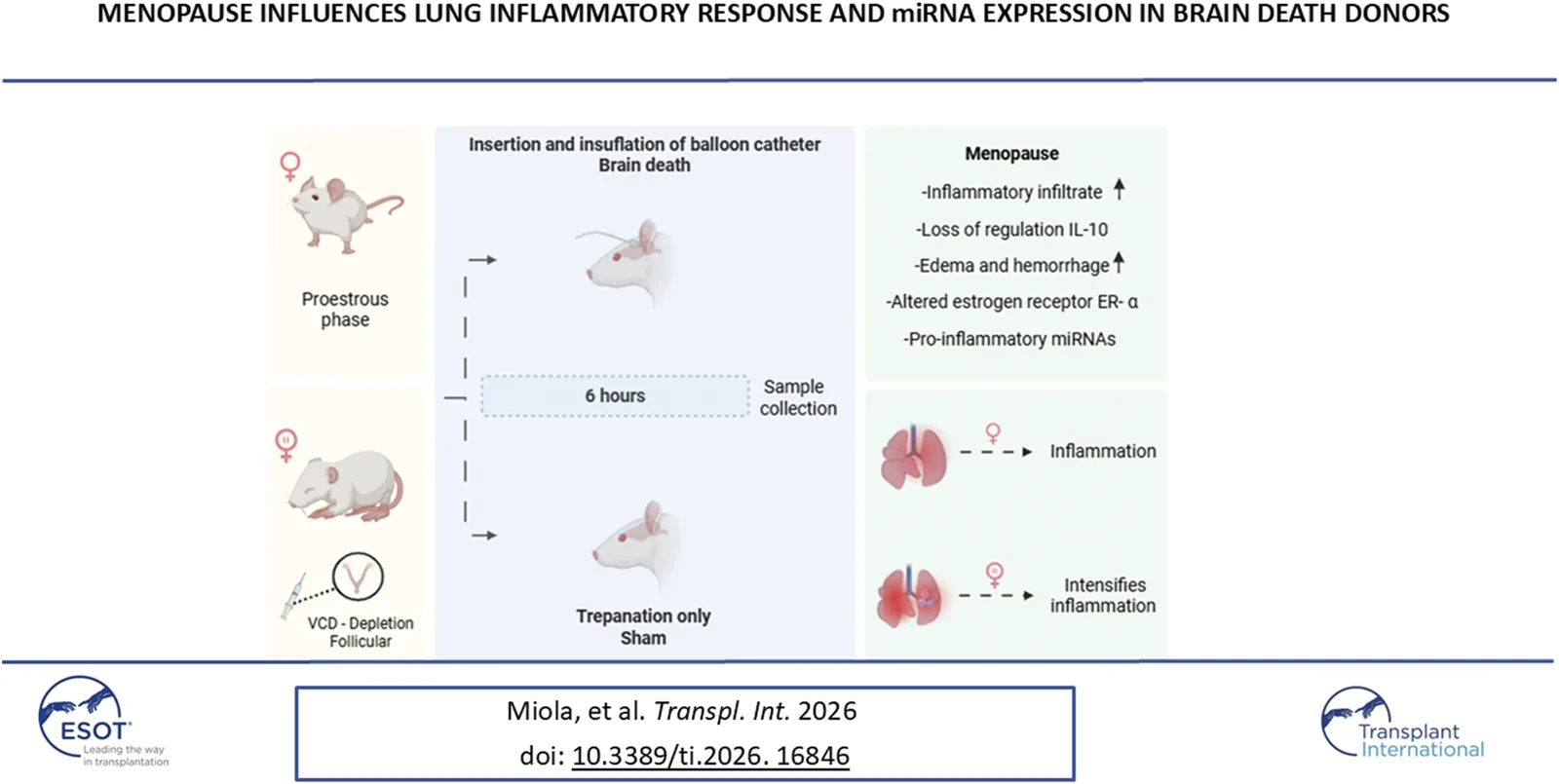

## Introduction

Organ transplantation is a fundamental approach for patients with advanced stages of various diseases, offering the possibility of prolonging and improving quality of life. However, current data highlight that the shortage of organs available for transplantation remains a global and growing challenge, aggravated by the aging population and the increasing demand of patients on waiting lists [1, 2]. According to the Global Observatory on Donation and Transplantation (GODT, 2025) out of 47.000 organ donors, 30% were over 60 years old and the average age of organ donors has been increasing over the years [3]. To respond to the high demand, the inclusion criteria were expanded, allowing the use of marginal organs, including those from older donors.

Brain death (BD), the main source of organ donation, triggers unregulated immunological and inflammatory changes that compromise the quality of organs intended for transplantation [1, 2, 4]. Among these, significant hormonal changes result from pituitary failure, with the lung being one of the organs most vulnerable to their effects and which may be associated with pulmonary edema after autonomic storm, such alterations may become a challenge for the success of lung transplantation [5, 6]. In the case of lung transplants, donors over 65 years of age are associated with shorter graft survival [7, 8].

Experimental studies, comparing males and females, confirm different responses to brain death. One such study demonstrated that female rats suffer greater lung injury after BD when compared to male rats [9]. Ferreira et al. highlighted important differences between the sexes to BD [10], mainly greater inflammation in females, but without impact on microcirculatory perfusion, an impact that was only documented in male animals [11, 12]. Such studies point to the importance of the acute reduction of female sex hormones after BD, after the loss of the hypothalamic-pituitary-ovarian axis resulting in greater inflammation in females.

Therefore, given the expansion of the criteria to include older donors and knowing that the donor’s sex and hormones influence the lung status, we investigated the effects of menopause and aging in rats subjected to BD, analyzing the release of inflammatory mediators and the influx of leukocytes into the lungs.

## Materials and methods

This study was approved by the Animal Research Ethics Committee of the Faculty of Medicine of the University of São Paulo under number (SDC) no 5635 23 037. All animals were handled in accordance with “Principles of Laboratory Animal Care” written by the National Society for Medical Research and the “Guide for the Care and Use of Laboratory Animals” published by the Institute of Laboratory Animal Resources from the National Institute of Health (NIH Publication No 86–23, revised 1996). Female Wistar rats (n = 48) were randomized into 4 groups: Menopause rats (Menopause BD; n = 8) and young rats (Young BD; n = 8) with both groups subjected to the BD procedure, and as controls, sham operation were used for both groups as well, the menopause (Menopause sham; n = 8) and young (Young sham; n = 8).

### Menopause model and aging

The chemical follicular depletion model was based on Mayer et al. (2002) [13]. Briefly, the rats (8 weeks old) received an intraperitoneal injection of 4-vinylcyclohexene diepoxide (VCD, 80 mg/kg, Merck, USA) diluted in corn oil for 6 weeks. After treatment, they were kept aging in the rodent vivarium for 10 weeks. At the end of the last week (170–180 days old), blood samples were collected for hormonal dosages and menopause confirmation. In addition, they were monitored weekly for weight and estrous cycle.

### Identification of the estrous cycle phases

The estrous cycle phase identification was performed by means of a vaginal smear using a Pasteur pipette with saline solution. The samples were placed on a slide and stained with 5 µL of crystal violet dye (5%), for identification by optical microscopy.

### Determination of serum hormone levels

Blood was collected from the animal’s tail vein and circulating hormone concentrations (estradiol, progesterone, corticosterone and follicle stimulating hormone (FSH)) were determined using Cayman kits (Cayman Chemical, USA) and MyBiosource (MyBiosource, USA). The protocols recommended by the manufacturers were used.

### Brain death model

The BD induction model was based on Breithaupt-Faloppa et al. [9]. Briefly, after intubation, anesthesia and vessel cannulation, a *Fogarty* ® 4F catheter (Baxter Healthcare, USA) was inserted in the intracranial space for rapid infusion of 400 µL of 0.9% NaCl. BD was confirmed by clinical criteria such as hypertensive peak pressure, bilateral mydriasis, apnea and absence of brainstem reflexes. Once confirmed, the use of isoflurane was discontinued. Animals in the sham group were subjected to surgical procedures without BD induction and remained anesthetized throughout the period.

### Determination of total and differential circulating leukocyte and platelet counts

At the beginning, at the third hour and at the end of the experiment, samples (20 μL of blood) were obtained, homogenized in diluent and subjected to automatic cell counting on a hematologic analyzer (BC-2800vet, Mindray, China).

### Concentration of IL-1β and IL-10 in serum and lung culture media

The determination of inflammatory mediators was performed using commercial Kits (Duo Set, R&D System®, USA). Serum samples were obtained 6 h after BD and lung tissue culture was established as previously described in Breithaupt-Faloppa et al. [9]. After 24 h media samples were collected. The mediator’s concentrations were expressed in pg/mL (serum) and pg/mL/mg of protein (media). Assays were conducted following manufacture specifications, and optical density was obtained using a spectrophotometer (SpectraMax® PLUS Microplate Reader, Molecular Devices, USA).

### Histopathological analysis of the lung

Lung fragments were fixed in formaldehyde solution (10%) for 24 h. The tissues were processed, embedded in paraffin, cut (4 µm) and the sections stained (hematoxylin/eosin). Conventional morphometric analyses were performed by two examiners blinded to the groups in order to evaluate hemorrhage and tissue edema. The sections were captured by a DS-Ri1 digital camera (Nikon, Japan), coupled to a microscope (Nikon). Five areas per image were extracted and exported in a format compatible with the NIS Elements Software (NIS-elements, Nikon, Tokyo, Japan).

### Bronchoalveolar lavage and bone marrow cell counts

After euthanasia, bronchoalveolar lavage (BAL) fluid was collected through the trachea with a syringe containing 5 mL of Phosphate Buffered Saline (PBS) injected into the airways. The BAL sample was centrifuged and the cell pellet was resuspended in 1 mL of PBS. The samples were subjected to automatic total and differential cell counting (BC-2800vet, Mindray, China). The bone marrow, the left femur was removed and a needle connected to a syringe containing 10 mL of PBS was introduced into the medullary canal. The sample were centrifuged and the cell pellet was resuspended in 1 mL of PBS. The samples were subjected to automatic total and differential cell counting (BC-2800vet, Mindray, China).

### MPO activity in lung tissue

The lung tissue samples were homogenized with 3 mL/g of hexadecyltrimethylammonium bromide (HTAB) and centrifuged and the ortho-dianisidine and H_2_O_2_ solutions were added. A sodium azide solution was added to stop the reaction. Using a spectrophotometer (Spectramax®, Molecular Devices, USA), absorbance was determined at 450 nm.

### Immunohistochemical investigation of adhesion molecules (ICAM-1), inducible nitric oxide synthase (iNOS), myeloperoxidase (MPO) and estrogen receptors (ER- α, ER-β and GPER)

Lung fragments were removed and inflated with a solution of OCT (optimal cutting temperature) solution, then immersed in hexane and frozen in liquid nitrogen. Serial sections (8 μm thick) were placed on silanized slides (StarFrost®, Knittelglass, Germany) and fixed in ice-cold acetone for 10 min. The slides were washed with TRIS-saline-tween-20 buffer solution (TBS-T) followed by blocking of endogenous peroxidase with H_2_O_2_ solution (2%) for 15 min at room temperature, and blocking of nonspecific sites with a protein buffer consisting of TBST and 2% bovine serum albumin (BSA) for 1 h at 37 °C. For the immunodetection of ICAM-1 and MPO (BosterBio, USA), iNOS (Abcam, USA), and estrogen receptors (Novus Biologicals, USA), primary antibodies diluted (1:100 and 1:200 for the estrogen receptors) in TBS-T/BSA were used. The sections were incubated overnight at 4 °C. The slides were washed with TBS-T and incubated with a 1:200 solution of anti-rabbit IgG secondary antibody associated with horseradish peroxidase (HRP) (BosterBio, USA) for 2 h at 37 °C. After another wash with TBS-T, the slides were incubated with 3-amino-9-ethylcarbazole (AEC) substrate solution for 30 min and counterstained with hematoxylin. The staining present in the tissue sections was recorded using an image acquisition system with a DS-Ri1 digital camera (Nikon, Japan) and analyzed using the NIS-Elements-BR software (Nikon). For the negative control, the samples were incubated with PBS instead of primary antibodies. The results analysis was expressed as stained area/vessel area (ICAM-1) and stained area/total area (MPO, iNOS, ER-α, ER-*β* and GPER).

### Real time PCR analysis of estradiol receptors and miRNA expression in lung tissue

RNA and miRNA extraction were conducted using a commercial kit (Mirvana, Applied Biosystems, CA, USA). Complementary DNA for the RNA and miRNA genes was synthesized using commercial kits: High-Capacity cDNA Reverse Transcription (Applied Biosystems, CA, USA) and TaqMan miRNA Reverse Transcription kit (Thermo Fisher, MA, USA), respectively. Real-time PCR (StepOnePlus; Applied Biosystems) for the estradiol receptor gene expression analysis was performed using the TaqMan primers (Applied Biosystems): GAPDH (RN01775763_g1), ER-α (RN01640372_m1), ER-β (RN00562610_m1), and GPER (Rn00592091_s1). For miRNA analysis, the following TaqMan primers (Applied Biosystems) were used: U6 snRNA (001973), 27a-3p (000408), 21a-3p (002493), 145-5p (002278), 155 (002571), 34c (000428), and 202-3p (462472). The targets were amplified following the following cycling conditions: 2 min at 50 °C, 10 min at 95 °C, 40 cycles of 15 s at 95 °C, and 1 min at 60 °C.

### Analysis of results

Data relating to hormones were subjected to the non-parametric Mean-Whitney test and the leukogram/platelets and blood pressure were analyzed of the three-way analysis of variance (ANOVA). The remaining results were analyzed using a two-way ANOVA, followed by the Benjamini, Kriger, and Yekutieli tests. Statistical analyses were performed using GraphPad Prism software version 10, and the results are expressed as mean ± standard error of the mean (SEM).

## Results

### Validation of the menopause model

**FIGURE 1:**
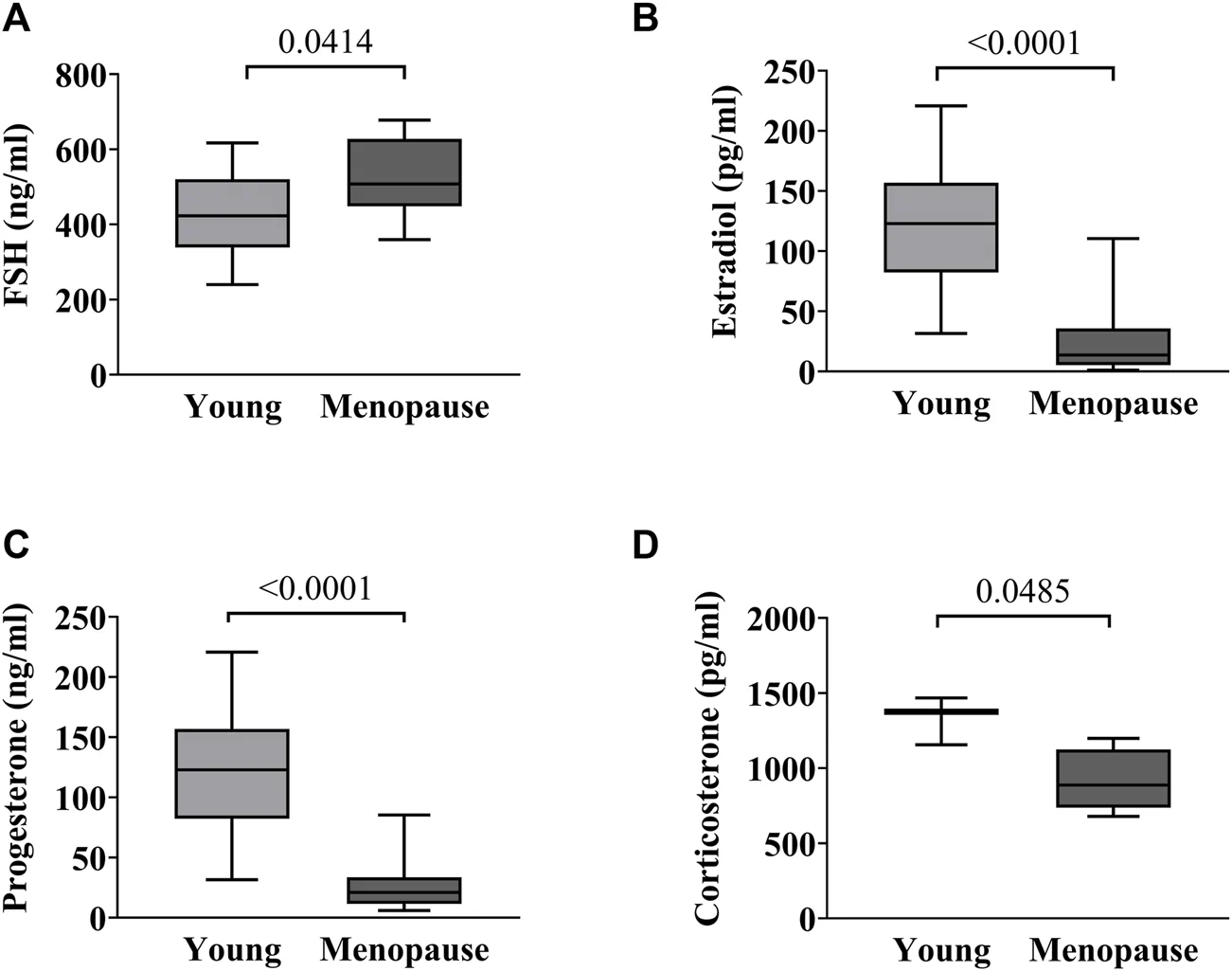
Characteristics of the female rats after menopause induction. **(A)** FSH serum concentrations; **(B)** Estradiol serum concentrations **(C)** Progesterone serum concentrations and **(D)** Corticosterone serum concentrations. Data are expressed as media±SEM from 16 animals.

Hormonal concentrations of FSH, progesterone, estradiol and corticosterone were measured in serum collected 1 week before the BD experiments. Rats in the menopause group showed an increase in FSH concentration and a significant decrease in progesterone, estradiol and corticosterone concentrations compared to young females (Figure 1).

### Analysis of hemodynamic parameters

**FIGURE 2:**
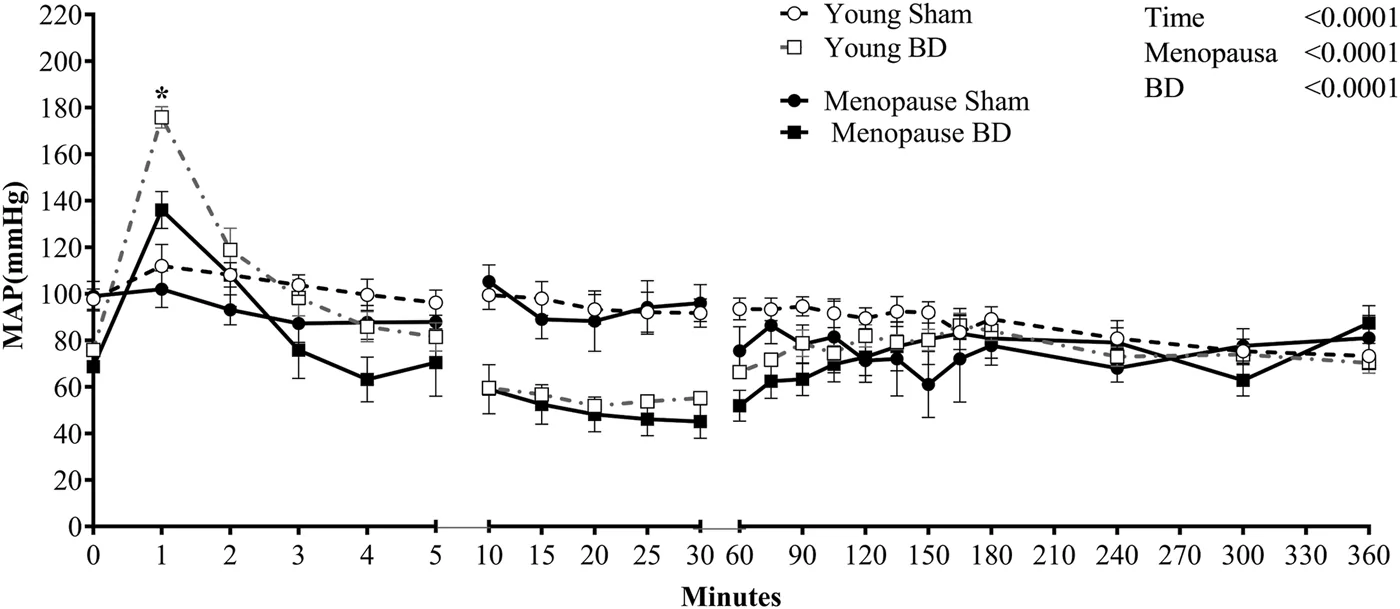
MAPs of Young-Sham, Young BD, Menopause Sham and Menopause BD female monitored from balloon catheter inflation (0) to the end of the experimental period (6 h). The data are presented as the mean ± SEM from 12 animals. * In relation the Menopause BD.

All the animals presented the hypertensive peak characteristic of the BD model, followed by a period of arterial hypotension of approximately 1 h, and by normalization of the blood pressure thereafter. The data show differences between the BD groups and their respective sham groups. BD rats showed a significant increase in mean arterial pressure (MAP) in the first minute after catheter inflation, compared to the sham group, and differences were observed between the young BD group and the menopause BD group. Menopause rats showed significantly lower PAM peak values compared to young animals, as shown in Figure 2.

### Determination of total and differential leukocyte counts

Total and differential leukocyte counts were analyzed, and a significant increase was observed in the total number of circulating leukocytes after BD surgical manipulation. The menopause sham group showed a significant increase, mainly in lymphocytes, at 6 h, compared to basal and the young sham group. In the young group, there was a predominantly granulocytes and monocytes increases just after 3 h, while in the menopause group, this increase only occurred after 6 h. In the 3-way ANOVA comparisons, there was a difference in relation to *time* in total leukocytes and in the cell types evaluated. In relation to *menopause*, there was a difference only in the total number of leukocytes. Regarding *BD*, there was a difference in all cell types but not in total leukocytes. In platelet analyses, a decrease in the number of platelets was observed after surgical manipulation (Table 1).

**TABLE 1:** Total and differential number of circulating leukocytes of rats from the young and menopausal group submitted or not (Sham) to brain death. Data represent the mean ± SEM.

WBC (cells/mm^3^)						PLT (×10^9^/L) Platelets
Groups		Total	Lymphocytes	Monocytes	Granulocytes	
Young Sham	0 h	9928.6±1643.1	6785.7±1274.4	400±72.4	2742.9±401.1	1069.4±59
	3 h	17471.4±1960.3*	4014.3±987.4	1014.3±290.7*	12442.9±1239.6*	708.1±165.1*
	6 h	19042.9±3250.1*	6057.1±1391.1	742.9±84.1	12242.9±3583.1*	709.6±124*
Young BD	0 h	12328.6±1190	7585.7±833.9	628.6±119	4114.3±496.9	1211.7±91.5
	3 h	11500±2058.2	4000±901.3	342.9±78.2^µ^	7157.1±1683.9^µ^	687.9±83.2*
	6 h	13085.7±2172.3	2985.7±689.2	414.3±50.8	9685.7±1616.1*	654.7±32.2*
Menopause Sham	0 h	7377.8±504.1	4833.3±285.8	377.8±49.4	2166.7±266.7	817.6±90^α^
	3 h	13133.3±2137.6	4833.3±968.7	755.6±157.3	5644.4±1620.7	695.8±95
	6 h	23311.14184.9*	15833.3±4432*^α^	1288.9±217.6*^α^	6200±1629.7^α^	819.2±79.4
Menopause BD	0 h	8357.1±737.7	5557.1±528.7	585.7±113.3	2321.4±231.9	913.6±52.9^α^
	3 h	11171.4±1740.8	5807.1±1072.1	642.9±83.7	5171.4±1480.2^α^	726.2±60.3
	6 h	15492.9±1425.2*^µ^	6807.1±.1064.6^µ^	1092.9±150.3^α^	7592.9±1535.5*	726.4±47.6
P time		<0.0001	0.0211	0.0015	<0.0001	<0.0001
P BD		0.5393	0.0322	0.0198	0.0006	0.2389
P Menopause		0.0140	0.0912	0.0885	0.3221	0.7281
P time × BD		0.0624	0.0010	0.0035	0.2848	0.0064
P Time × Menopause		0.0169	0.0049	0.0100	0.2553	0.2635
P BD × Menopause		0.9211	0.3731	0.1889	0.1672	0.9081
P Time × Menopause × BD		0.5521	0.2717	0.3531	0.3421	0.9016

* In relation the respective Basal; α in relation a young; µ in relation the respective sham.

### Analysis of the hemorrhagic profile and edema in the lungs

**FIGURE 3:**
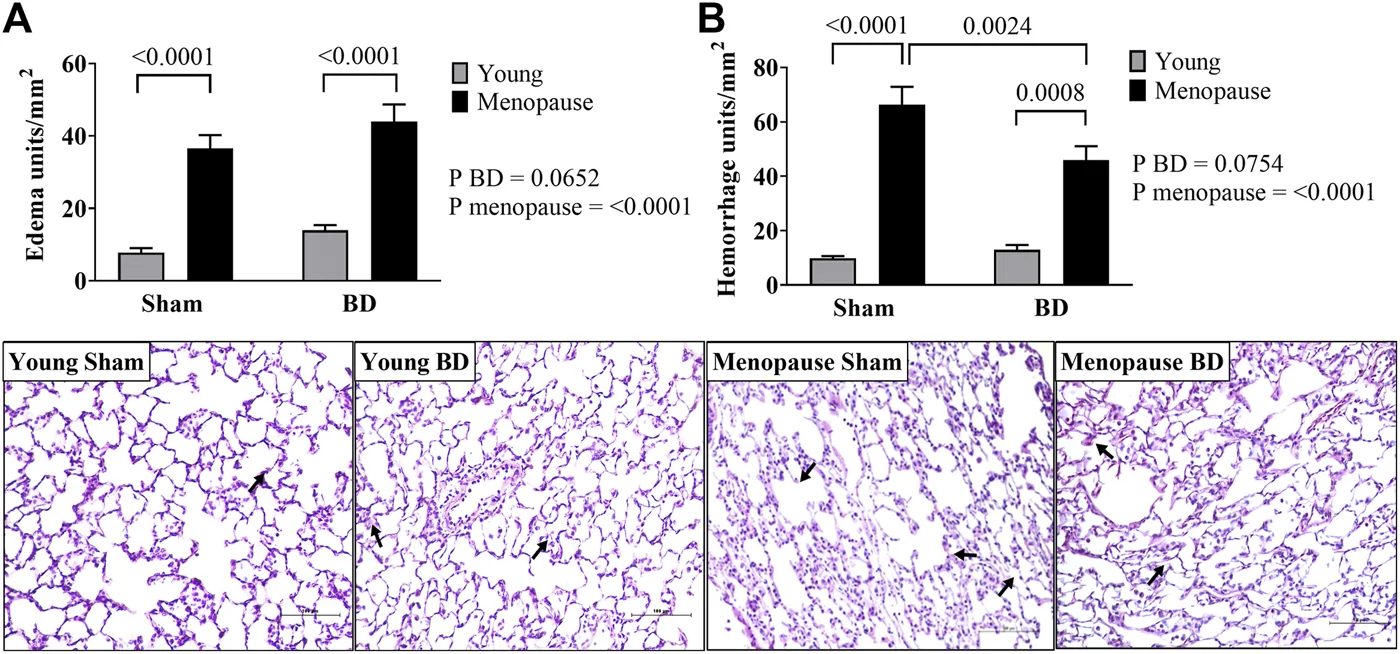
Histological analysis of lung sections (HE). **(A)** edema and **(B)** hemorrhage from young or menopause rats subjected or not (Sham) to BD. Data represents the mean ± SEM from 8 animals. Arrows show higher intensity of pulmonary hemorrhage in menopause sham animals compared to other groups.

As observed in Figure 3, menopause rats, independent of BD, presents greater edema and hemorrhage compared to young rats (Sham and BD).

### Cell quantification in bronchoalveolar lavage fluid and bone marrow

**FIGURE 4:**
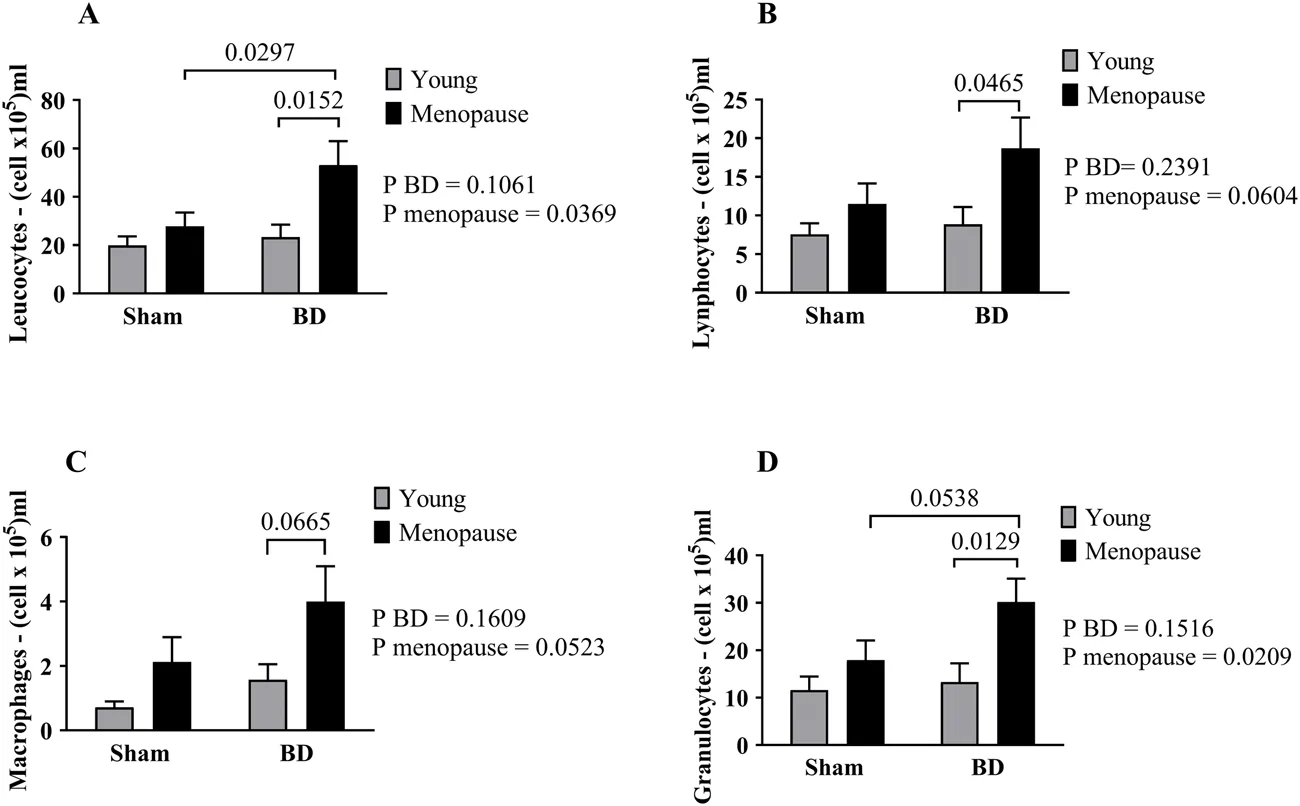
Total number of cells **(A)** leucocytes; **(B)** lymphocytes, **(C)** macrophages and **(D)** granulocytes from rats from the young and menopause group submitted or not (Sham) to BD. Values represent the mean ± SEM from 8 animals.

The data show a significant increase in all cell types in bronchoalveolar lavage fluid in menopause BD rats compared with young BD. In addition, there was a higher number of lymphocytes and granulocytes in the menopause BD than their respective sham, as shown in Figure 4. The results of bone marrow counts do not indicate any differences between groups as shown at the Table 2.

**TABLE 2:** Total number of bone marrow cells from young and menopause rats subjected or not (sham) to brain death. Values represent the mean ± SEM.

Bone marrow (10^6^/mL)	
Young sham	69.071 ± 8.981
Young BD	68.071 ± 10.261
Menopause sham	60.044 ± 19.222
Menopause BD	60.679 ± 19.677
*P BD*	0.9745
*P menopause*	0.1580

### Concentration of serum and lung culture (explant) inflammatory mediators

In Table 3 the quantification the inflammatory mediators in the serum and lung explant is presented. There was a significant increase in serum IL-10 concentration in the young BD compared to the respective sham group, however this response was not observed in menopause BD.

**TABLE 3:** Concentration of serum mediators (pg/mL) and explant (pg/mg) of rats from the young and menopause group submitted or not (Sham) to brain death. Serum 6 h and lung culture explant 24 h. Data presented as the mean ± SEM.

Inflammatory mediators				
​	Serum		Explant	
Groups	IL-1	IL-10	IL-1	IL-10
Young sham	3.03 ± 0.18	3,137.11 ± 1,639.59	88.38 ± 100.53	4,197.70 ± 2,306.19
Young BD	3.16 ± 0.72	8,687.55 ± 2,515.96*	239.05 ± 209.40	2,877.78 ± 2,400.87
Menopause sham	4.04 ± 0.60	2,172.52 ± 1,539.55	46.02 ± 63.80	1,071.33 ± 375.90^α^
Menopause BD	3.96 ± 0.65	2,161.83 ± 2047.88^α^	180.64 ± 243.93	1,418.66 ± 1,314.57^α^
*P BD*	0.9762	0.1724	0.0613	0.4138
*P menopause*	0.3086	0.0693	0.4972	0.0005

* in relation to Young sham; α in relation to Young BD.

### Evaluation of the presence of leukocytes in lung tissue

**FIGURE 5:**
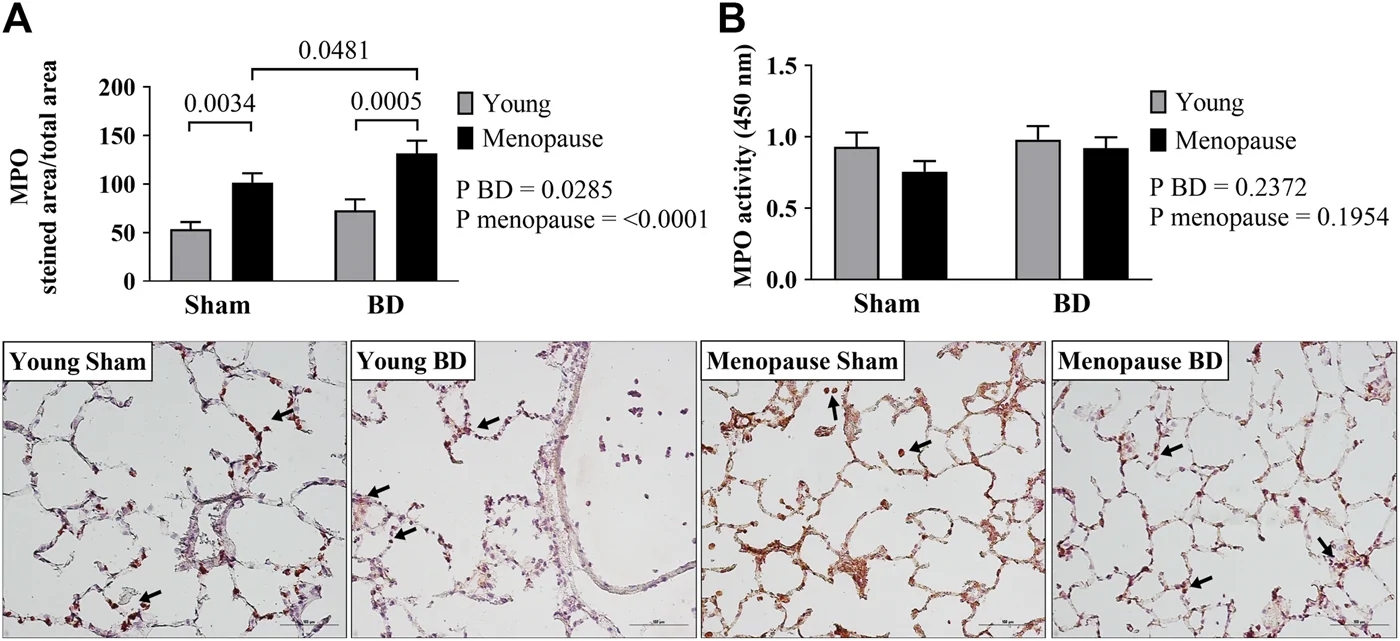
**(A)** Number of cells stained for MPO in tissue sections from 6 animals and **(B)** Myeloperoxidase (MPO) activity from 8 animals in lung tissue samples from young or menopause rats submitted or not (Sham) to BD. Data represents the mean ± SEM. Arrows show higher number of cell stained for MPO in menopause group in relation to young.

No significant differences were observed in the analysis of MPO activity (Figure 5B). Additionally, quantification of lung MPO positive stained cells was performed and the results showed an increase in these cells in menopause rats, in both the sham and BD groups (Figure 5A).

### Quantification of ICAM-1 and iNOS

**FIGURE 6:**
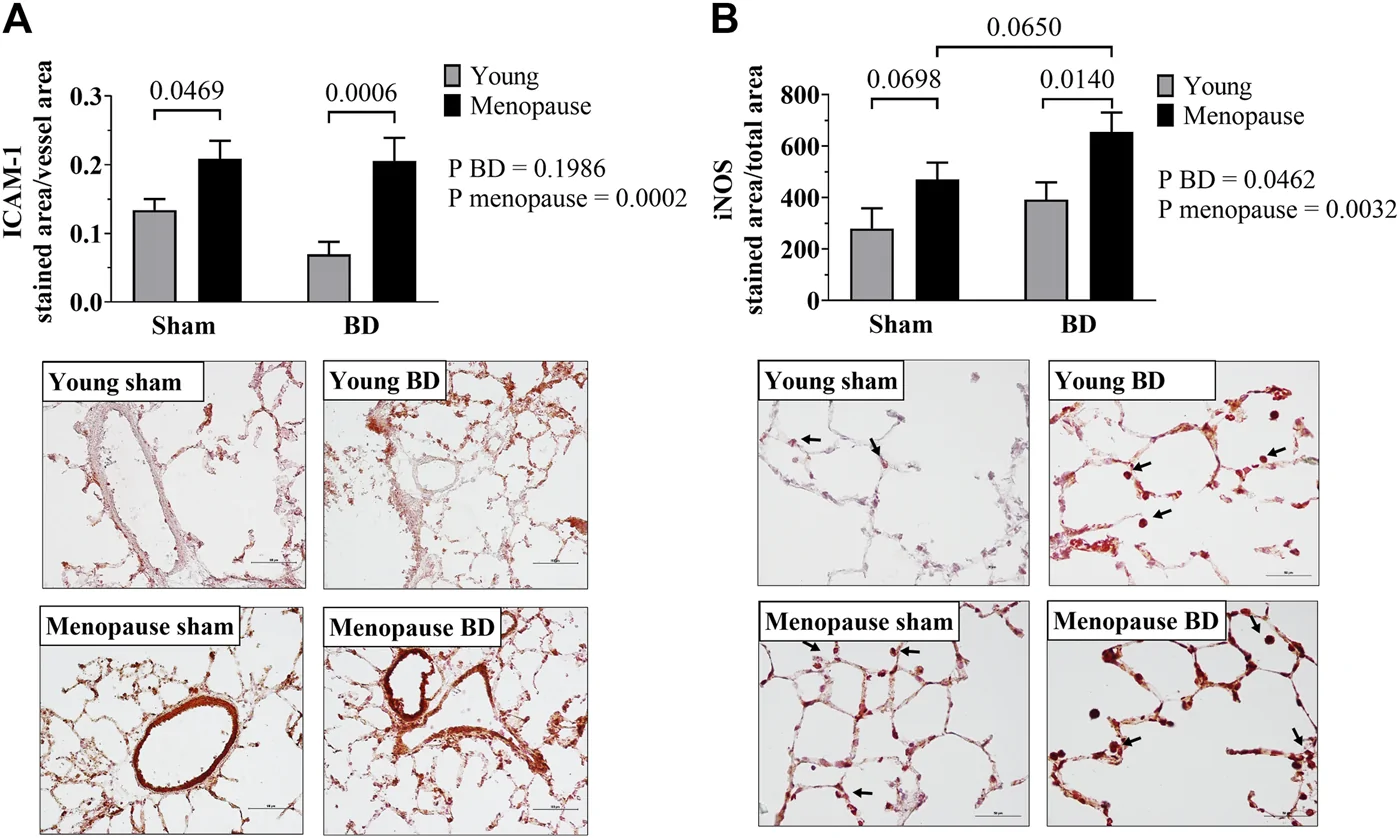
**(A)** Expression of intercellular adhesion molecule (ICAM)-1 and **(B)** inducible nitric oxide synthase (iNOS) in lung sections from young or menopause rats subjected or not (Sham) to BD. Representative photomicrographs of the immunohistochemical reaction. Data represents the mean ± SEM from 5 animals.

The data indicate an increase in ICAM-1 expression in menopause rats, independent of BD (Sham and BD) (Figure 6A), and a higher number of iNOS positive cells were also observed in the menopause group compared to young rats (Figure 6B).

### Quantification of estradiol receptors in lung tissue

**FIGURE 7:**
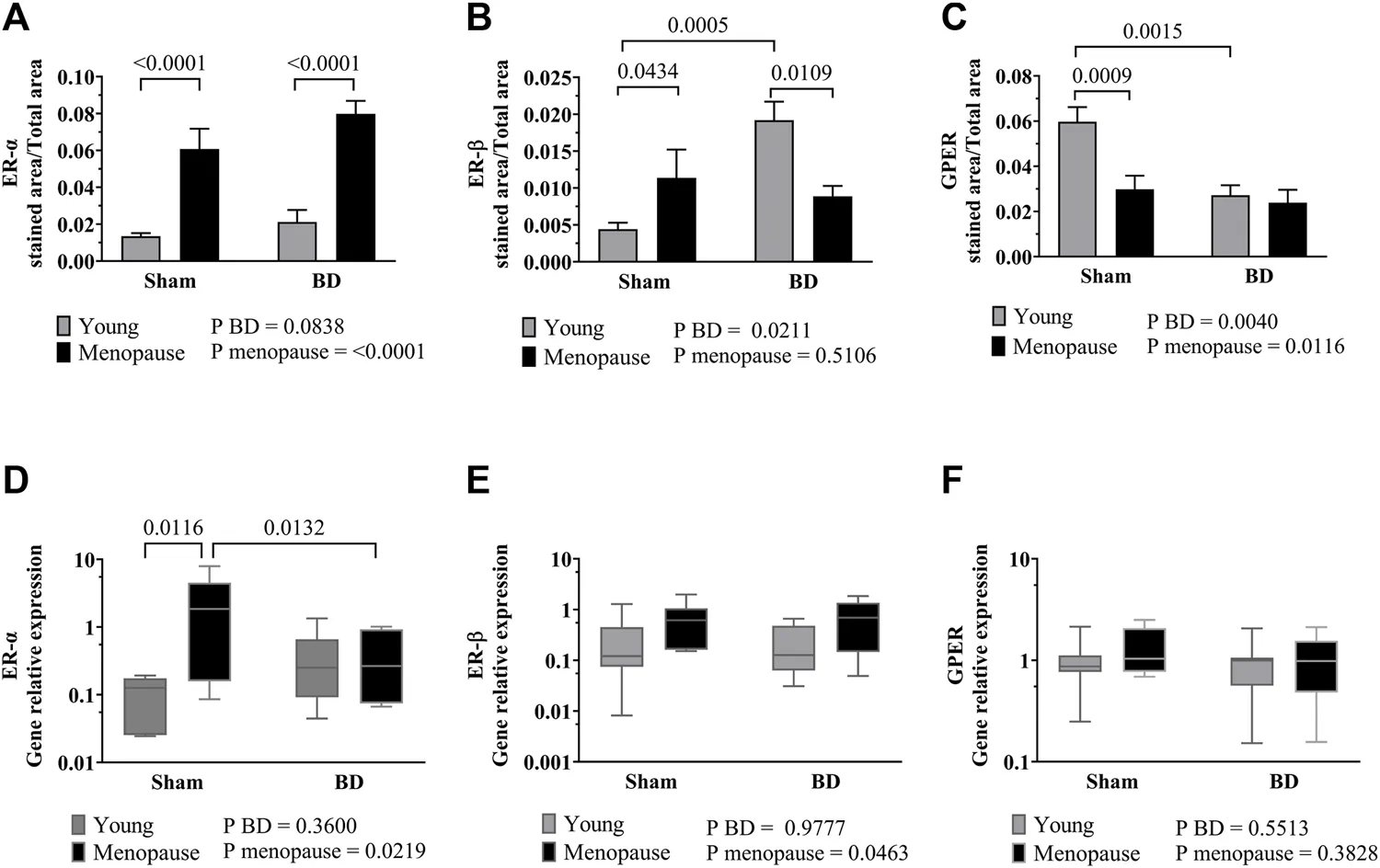
Protein and gene expression of the **(A,D)** estradiol receptor α (ER-α), **(B,E)** estradiol receptor β (ER-β) and **(C,F)** protein G associated estradiol receptor (GPER) in lung sections from young or menopause rats subjected or not (Sham) to BD. In **(A–C)**, data represents the mean ± SEM and in **(D–F)**, data are expressed as median and 95% percentile interval (n = 8 animals).

Estradiol receptors expression analyses in lung tissue were also performed, as shown in Figure 7. A significant increase in ER-α expression was observed in menopause rats (BD and Sham) when compared to young rats. In addition, the ER-α gene expression is upregulated in menopause sham rats compared and young sham rats, being reduced by BD. The data also indicates a reduction in GPER expression in menopause rats (Sham and BD) and in young rats subjected to BD. The same was not observed when evaluating the gene expression of this receptor. Differently, in the analysis of ER-β expression, there was an increase in young BD rats compared to their respective sham, with a lower expression of this receptor in menopause BD rats.

### miRNA receptors in lung tissue

**FIGURE 8:**
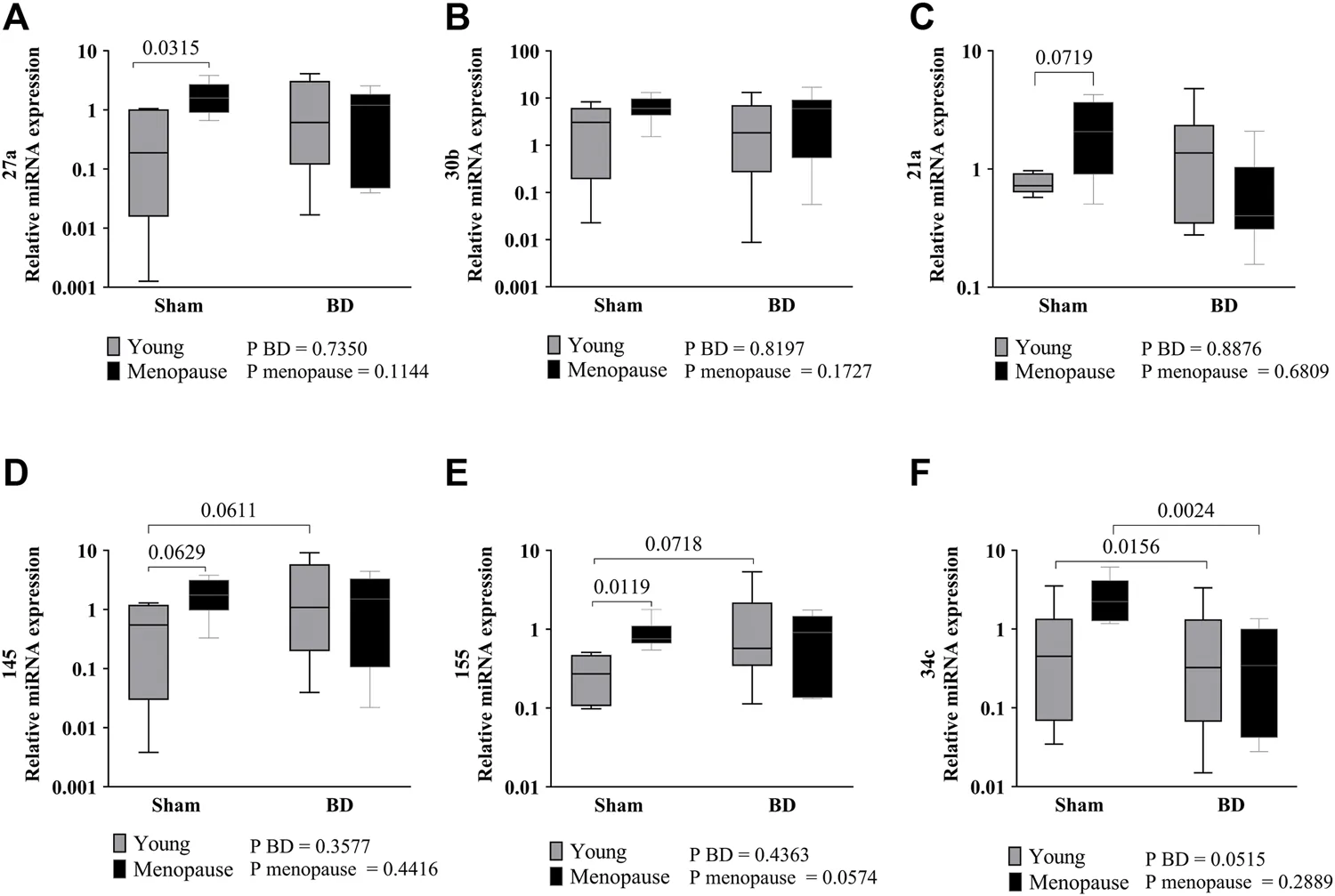
Analysis of miRNA expression in lung tissue from young or menopause rats subjected or not (Sham) to BD. **(A)** miRNA 27a; **(B)** miRNA 30b; **(C)** miRNA 21a; **(D)** miRNA 145; **(E)** miRNA 155 and **(F)** miRNA 34c. Data expressed as median and 95% percentile interval from 7 animals. Relative gene expression analysis and miRNA were normalized using endogenous control, and relative expression was determined using naive control (non-manipulated rats).

miRNAs expression were analyzed in lung tissue, and an upregulation of miRNAs 27a, 21a, 145, 155, and 34c was observed in menopause sham rats compared to young sham. The miRNAs 145, 155, and 34c were upregulated in young BD rats compared to their respective sham. In addition, a downregulation of miRNA 34c was observed after BD (Figure 8).

## Discussion

Donor inclusion criteria have been expanded across organ transplantation to address organs shortage and increase the transplant opportunities. This expansion includes accepting organs from older donors, and, when the donor is female, it is important to consider menopause. Menopause is accompanied by several physiological changes, including those in the immune system. In order to understand the effects of menopause on lung donors, we submitted menopause rats to BD, comparing them with young animals. Most studies still focus on changes occurring in male donors; therefore, it is important to emphasize that this study specifically investigated the effects of menopause in organ donors, a group that has been understudied but growing steadily over the years. In this context, we chose to use an experimental model of menopause that better reflects the hormonal fluctuations observed clinically in women [13]. The association of BD with menopause exacerbated lung inflammation, reinforcing the importance of considering the woman’s age and cycle stage when evaluating lung donors.

Previous clinical and experimental studies identified the acute reduction in female sex hormones following BD [9, 14]. This reduction is related to increased systemic and lung inflammation in young female rats, and treatment with estradiol is able to reduce leukocyte infiltration and edema formation [15]. Here, menopause led to even higher leukocyte infiltration and edema, accompanied by an increase in all cell types in the BAL and increased MPO expression. In addition, in the menopause group, the leukocyte response was more intense and late compared to young animals. These results emphasize the greater mobilization of leukocytes to the lung parenchyma and suggest a compromise in the regulatory mechanisms of inflammation. In this sense, clinical studies have demonstrated that the menopause period is characterized by a pro-inflammatory state, induced mainly by the reduction of female sex hormones, and that the estrogen deficiency marked by menopause can impact the leukocyte subpopulation [16–18].

Our data indicate an increase in iNOS protein expression in lung tissue in the menopause group, as well as an increase in ICAM-1 expression, which may lead to greater leukocyte adhesion and migration to lung tissue. iNOS may contribute to an imbalance in nitric oxide (NO) production, which can intensify the inflammatory process and favor increased vascular permeability [19]. In addition, higher lung hemorrhage was observed in the menopause sham group. These animals have reduced hormone concentrations due to the induction of menopause and were maintained mechanically ventilated. One study showed that the reduction of sex hormones leads to the formation of new vessels and the loss of vascular protection conferred by estrogen [20]. These new vessels could, therefore, be more prone to injury and rupture, mainly to hemorrhage. This indicates that the menopause period itself can compromise vascular integrity. Previous study by the group using ovariectomized young rats [21] differ from this, specially by the time of hormonal reduction; here, the aging factor was added to the protocol, once menopause is a condition associated with higher age.

Since estrogen deficiency is associated with mechanisms that regulate inflammation, we investigated serum inflammatory mediators. IL-10 is a cytokine that helps to regulate the inflammatory response [22]. In our study, we observed increased IL-10 production in young rats, an effect not observed in the menopause group. Furthermore, we performed cytokine quantifications in lung explant culture medium (24 h), which reinforced the idea that there is a loss of regulation in the production of this cytokine. Clinical studies suggest that during menopause, women have higher production of pro-inflammatory cytokines and lower production of anti-inflammatory cytokines [22–24].

Considering the modulating role of estradiol in the inflammatory response, we performed analyses of estradiol alpha, beta, and GPER receptors. Genetic and protein alterations were observed with increased expression of ER-α in menopause rats, possibly a compensatory mechanism for the loss of the hormone. This receptor is strongly associated with reproductive function, vascular protection, and multiple mechanisms [25]. Regarding ER-β and GPER receptors. ER-β is associated with the modulation of the immune response and shows high expression in the lungs, while GPER, being a membrane receptor with rapid and non-genomic signaling, may not show detectable changes at the gene level [26–28].

To better understand the mechanisms involved in the inflammatory response observed primarily in menopause rats, we performed a miRNA screening based on their association with inflammatory processes, oxidative stress, apoptosis, senescence, and vascular function. We observed that miRNA 27a-3p, already upregulated in the menopause sham group, is associated with modulation of macrophage function and may suppress the anti-inflammatory cytokine IL-10 via TGF-β, being largely studied as a potential therapeutic target for the treatment of inflammatory conditions [29, 30]. The miRNAs 21a, 145 and 155 were also found upregulated in the menopause sham group in comparison to young sham indicating their modulation by menopause process. The miRNA 21-5p is highly expressed in monocytes/macrophages and takes an important role in cardiovascular diseases, cancer, and immune response, in addition to its role in the process of chronic inflammation associated with aging (inflammaging) [31]. It can modulate the inflammatory response through direct and indirect activities in the NF-κB and NLRP3 pathways, depending on the context [32, 33]. Like miRNA 21a, miRNA 155 is strongly associated with inflammaging, and due to its central role in the innate and adaptive immune response, it has also been altered during menopause, reinforcing its pro-inflammatory context [34, 35]. miRNA 145, is associated with vascular changes and leukocyte adhesion, in addition to its pro-inflammatory role [36]. This may contribute to the increased ICAM-1 observed in our results. In vascular smooth muscle cells from patients with diabetes type 2, the secretion of pro-inflammatory cytokines has been associated with elevated miRNA levels, suggesting a role in vascular senescence [37]. The miRNA 34c, associated with apoptosis and senescence processes [25], showed upregulation in the menopause group compared to young rats and controls. Studies show that miRNAs are related to menopause, since the hormonal decline alters post-transitional regulatory mechanisms of genes involved in the regulation of the immune and inflammatory response. Our findings highlight the role of miRNAs in the context of BD induced lung inflammation in menopause females.

It is important to highlight that, from a clinical point of view, menopause in humans occurs alongside aging, which makes this model translationally relevant. The follicular depletion model using VCD represents one of the closest models to the human menopause transition. However, a limitation of the present study is that it does not allow for a complete differentiation between the effects of menopause and aging, since, after follicular depletion, the animals continue to age naturally. Therefore, some of the observed inflammatory effects may be related not only to the loss of ovarian hormones but also to processes associated with inflammaging. Additionally, the animals used do not correspond to such an advanced stage of aging, suggesting that senescence processes may still be in their initial phases. Future studies including the evaluation of hormonal rescue with estradiol or estrogen receptor modulators may help to further elucidate the mechanisms related to menopause and aging.

Taken together, our study reinforces the idea that female sex hormones have a significant influence on modulating the inflammatory state generated by BD. In addition, the results show that menopause intensifies lung inflammation, characterized by increased inflammatory infiltrate, alterations in nitric oxide synthase and adhesion molecules, and, most importantly, by the loss of IL-10 regulation. Considering the increasing number of female donors in menopause, our findings reinforce the relevance of investigating therapeutic strategies targeted at this group.

## Data Availability

The raw data supporting the conclusions of this article will be made available by the authors, without undue reservation.
